# Supplementary material for: miRNome and Proteome Profiling of Small Extracellular Vesicles Secreted by Human Glioblastoma Cell Lines and Primary Cancer Stem Cells
Source: Biomedicines. 2022 Aug 4;10(8):1886. doi: 10.3390/biomedicines10081886 (PMC9405730; doi:10.3390/biomedicines10081886)
Supplement: Supplementary file 1 [file biomedicines-10-01886-s001.zip › biomedicines-1822438-supplementary.pdf]

## Supplementary Figures

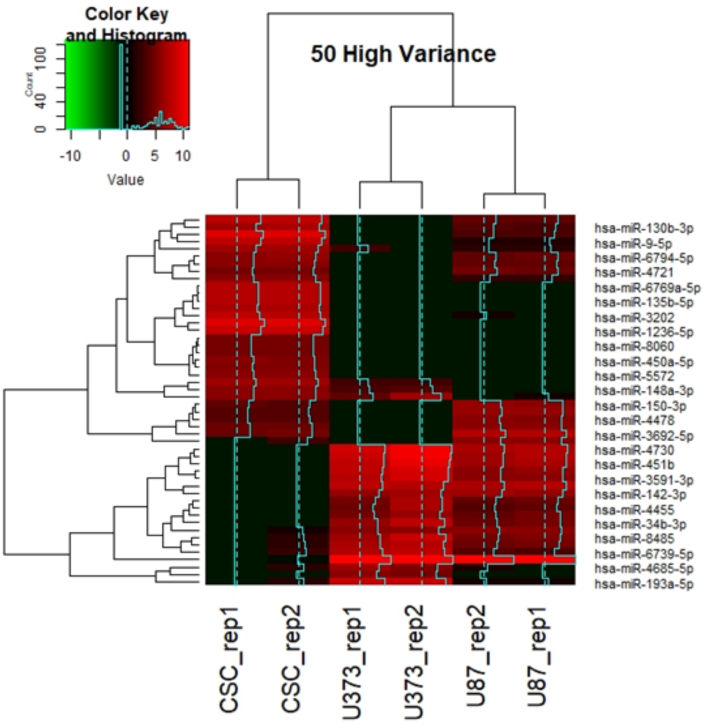


**Figure S1**. Hierarchical clustering heat-map of the first 50 high-variance miRNAs.

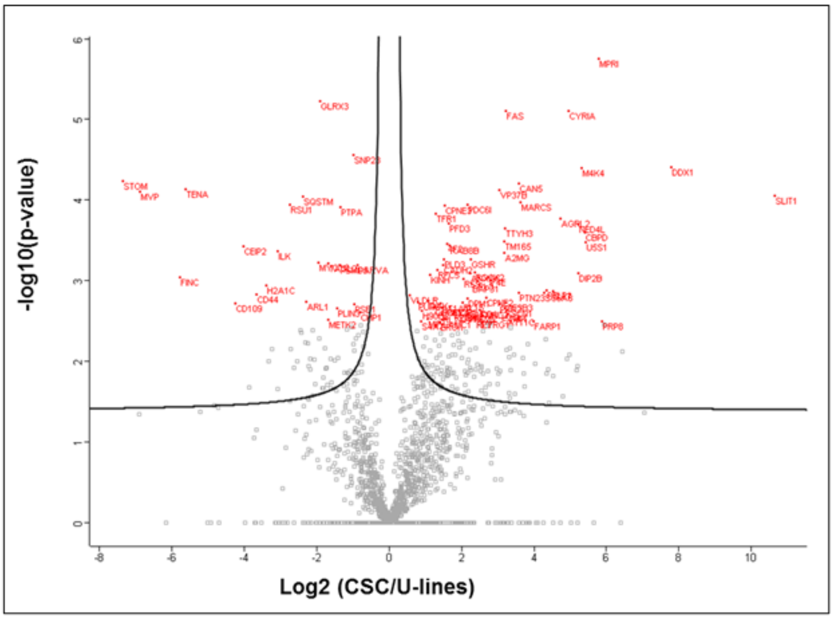


**Figure S2**. Volcano plot showing differentially expressed proteins in CSC-sEV samples versus GBM-sEVs (U373 and U87, U-lines). At FDR < 0.05, 249 proteins get significant differences (see Suppl. Table 5).
